# Expression of non-secreted IL-4 is associated with HDAC inhibitor-induced cell death, histone acetylation and c-Jun regulation in human gamma/delta T-cells

**DOI:** 10.18632/oncotarget.11462

**Published:** 2016-08-20

**Authors:** Jaydeep Bhat, Justyna Sosna, Jürgen Fritsch, Elgar Susanne Quabius, Stefan Schütze, Sebastian Zeissig, Ole Ammerpohl, Dieter Adam, Dieter Kabelitz

**Affiliations:** ^1^ Institute of Immunology, Christian-Albrechts-University, Kiel, Germany; ^2^ Department of Otorhinolaryngology, Head and Neck Surgery, Christian-Albrechts-University, Kiel, Germany; ^3^ Department of Internal Medicine I, Christian-Albrechts-University, Kiel, Germany; ^4^ Institute of Human Genetics, University Medical Center Schleswig-Holstein Kiel, Christian-Albrechts-University, Kiel, Germany; ^5^ Current address: Department of Molecular Biology and Biochemistry, University of California-Irvine, Irvine, CA, USA; ^6^ Current address: Department of Medicine I, University Medical Center Dresden, Technical University Dresden, Dresden, Germany; ^7^ Current address: Center for Regenerative Therapies Dresden (CRTD), Technical University Dresden, Dresden, Germany

**Keywords:** IL-4, apoptosis, necroptosis, HDAC inhibitors, valproic acid, Immunology and Microbiology Section, Immune response, Immunity

## Abstract

Previously, the expression of a non-secreted IL-4 variant (IL-4δ_13_) has been described in association with apoptosis and age-dependent Th2 T-cell polarization. Signaling pathways involved in this process have so far not been studied. Here we report the induction of IL-4δ_13_ expression in human γδ T-cells upon treatment with a sublethal dose of histone deacetylase (HDACi) inhibitor valproic acid (VPA). Induction of IL-4δ_13_ was associated with increased cytoplasmic IL-4Rα and decreased IL-4 expression, while mRNA for mature IL-4 was concomitantly down-regulated. Importantly, only the simultaneous combination of apoptosis and necroptosis inhibitors prevented IL-4δ_13_ expression and completely abrogated VPA-induced global histone H3K9 acetylation mark. Further, our work reveals a novel involvement of transcription factor c-Jun in the signaling network of IL-4, HDAC1, caspase-3 and mixed lineage kinase domain-like protein (MLKL). This study provides novel insights into the effects of epigenetic modulator VPA on human γδ T-cell differentiation.

## INTRODUCTION

Based on TCR gene rearrangement, human T-cells are classified as αβ T-cells (about 90-95%) or γδ T-cells (about 5-10% of total CD3). Most peripheral blood γδ T-cells express Vδ2 paired with Vγ9 whereas intestinal resident γδ T-cells usually express Vδ1. Human Vγ9Vδ2 T-cells recognize pyrophosphate intermediates of the non-mevalonate and the mevalonate isoprenoid biosynthesis pathways that are produced by microbes and tumor cells, respectively [1, 2]. γδ T-cells display potent anticancer activity against leukemias/lymphomas and various epithelial tumor cells, and thus have emerged as a promising target for cancer immunotherapy [3, 4]. Apart from their antitumor response, they possess a surprising functional plasticity which includes follicular B-cell helper activity but also regulatory activity. T-cell subset differentiation is governed by a set of specific transcription factors and the local cytokine milieu. Under appropriate microenvironmental conditions, human γδ T-cells can thus differentiate *in vitro* into Th1, Th2, Th17, Tfh and Treg lineages [5-8].

Induction of cell lineages and functional responses to microenvironmental stimuli trigger subsequent intracellular signaling networks. Mechanisms controlling cellular function and expression of such signaling molecules are mainly associated with chromatin remodeling and histone modifications [9]. Histone modifications like acetylation are directed by histone-modifying enzymes including histone acetyl transferase (HAT) and histone deacetylase (HDAC), sharing potential cross-talk between different modifications [10]. In addition, HDACs are reported to control cellular functions at the epigenetic level [9, 10]. More than 18 HDACs have been shown to have non-redundant functions. They are primarily grouped as class I (HDAC1, 2, 3, 8), class II (HDAC4, 5, 7, 9), class IIa (HDAC6, 10), class IV (HDAC11; sharing class I and II deacetylases) and NAD^+^-dependent class III (sirtuins) [11]. Valproic acid (VPA), inhibitor of HDAC (HDACi), has been widely used in the clinic as anticonvulsant for the treatment of epilepsy but is also explored as anticancer agent [12, 13]. VPA is a FDA-approved short-chain fatty acid inhibitor that targets class I HDAC [14].

We have previously reported that VPA treatment at “toxic” concentration (5 mM) results in selective survival of αβ T-cells over γδ T-cells. Also, treatment of human γδ T-cells with VPA-induced genome-wide histone H3 acetylation and the differential modulation of a restricted set of surface markers only on surviving γδ T-cells in comparison to αβ T-cells [15]. These findings led us to further investigate the molecular consequences of VPA treatment on short-term expanded human γδ T-cells. Our present study shows strong induction of a non-secreted form of IL-4 (IL-4δ_13_). Previously, this non-secreted form of IL-4 has been shown to be associated with increased CD4 T-cell apoptosis in HIV-infected individuals and with a Th2 precursor phenotype in infants [16, 17]. While inhibitors of apoptosis and necroptosis had only minor effects on VPA-induced cell death, they prevented induction of IL-4δ_13_ and in combination inhibited H3 acetylation, yet up-regulated c-Jun protein expression. Thus, this study reveals a signaling network upon VPA treatment with relevance for the functional plasticity of γδ T-cells.

## RESULTS

### HDACi induces IL-4δ_13_ in human γδ T-cells

**Figure 1:**
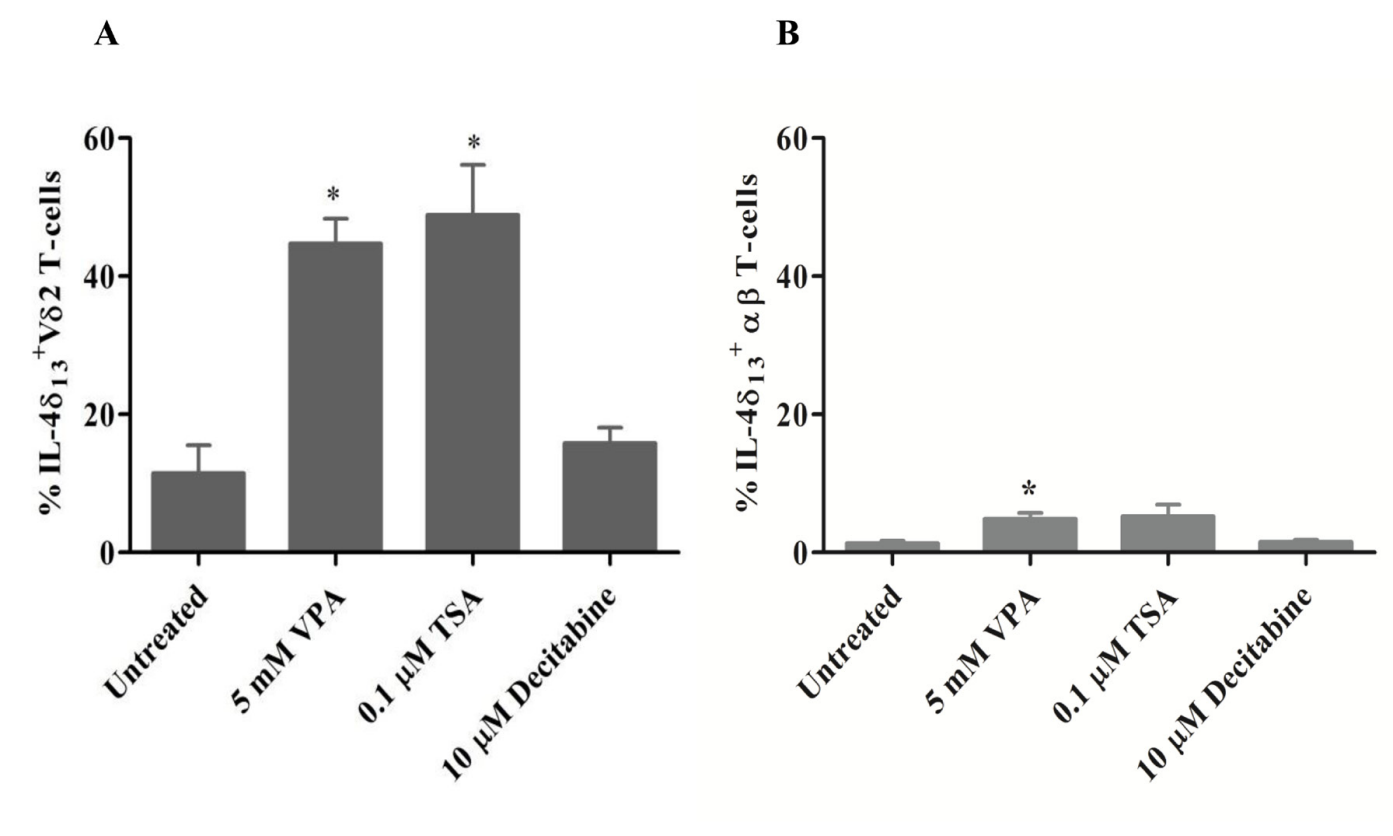
Induction of IL-4δ_13_ by HDACi treatment in human T-cells γδ T-cell lines generated from PBMC stimulated for 12 d with zoledronate and IL-2 (**A**) or αβ T-cell lines generated from PBMC with a staphyloccal enterotoxin mixture (**B**) were treated for 24 hrs with the indicated concentrations of VPA, TSA, or decitabine. Thereafter, T-cells were subjected to FACS analysis of T-cells co-expressing intracelluar IL-4δ_13_. Dead cells were excluded based on live/dead fixable far-red dye staining. Data represent mean ± S.E. of 3 independent experiments. Statistical significance shown by * indicates p-values <0.05.

Epigenetic modifiers are known to modulate transcription factor and intracellular cytokine expression [18, 19]. Here we analyzed intracellular IL-4 expression in activated and proliferating human γδ T-cells cultured for 24 hrs in the presence of HDACi. We used anti-IL-4 mAb 8D4-8, which specifically detects a non-secreted isoform with a 13 bp deletion (IL-4δ_13_) that has been associated with apoptosis and age-dependent Th2 differentiation [16, 17, 20]. As shown in Figure 1A, treatment with HDACi VPA and trichostatin A (TSA), but not with the hypomethylating agent decitabine, stimulated significant expression of IL-4δ_13_ in surviving Vδ2 T-cells. In comparison to Vδ2 T-cells, only a very small amount of IL-4δ_13_ expression was induced in surviving αβ T-cells (Figure 1B).

**Figure 2:**
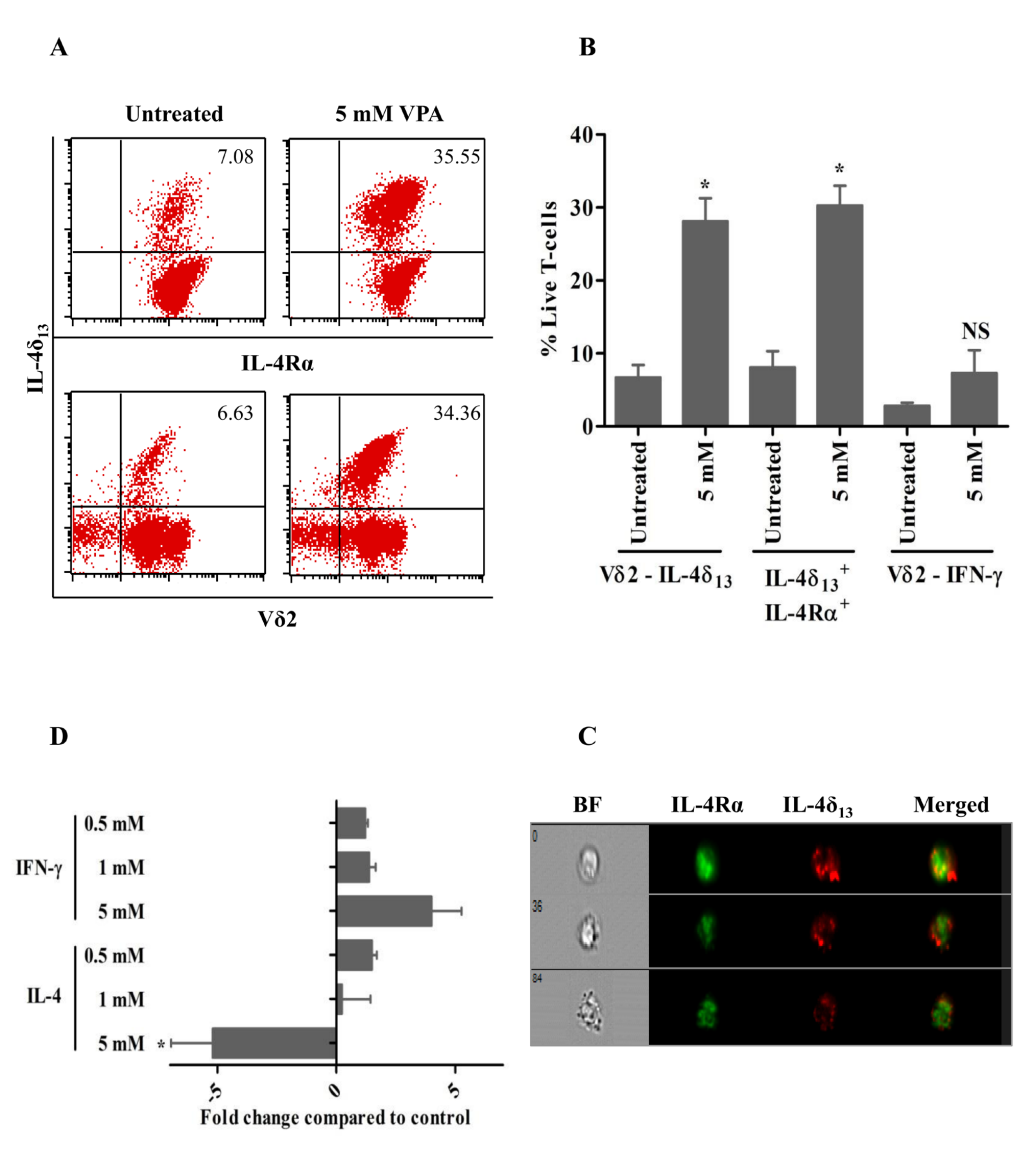
VPA-regulated expression of intracellular cytokines in human γδ T-cells γδ T-cell lines generated from PBMC stimulated for 12 d with zoledronate and IL-2 were treated for 24 hrs with 5 mM VPA, and monensin was added for last 4 hrs. Thereafter, intracellular expression of IL4δ_13_, IL-4Rα and IFN-γ was determined by FACS. (**A**) Dot plot analysis of a representative experiment. The upper panel row shows IL4δ_13_ versus IL-4Rα, lower panel row shows IL4δ_13_ and Vδ2 expression. A combined gate was set on FSC versus SSC and live/dead fixable far-red dye negative population.(**B**) Summary of 3 independent experiments. (**C**) γδ T-cells were also analyzed by ImageStream cytometry for possible co-localization of IL-4δ_133_ with IL-4Rα. Images represented are live, single cell populations of 5 mM VPA-treated γδ T-cells analyzed within focus, with marked column as bright field (BF), FITC-labelled IL-4Rα, PE-labelled IL-4δ_13_ and both fluorochromes merged. Representative single cells from three independent experiments are shown. (**D**) γδ T-cells obtained as above were treated for 24 hrs with the indicated concentrations of VPA. Thereafter, mRNA expression of IFN-γ and IL-4 was quantified by real time PCR. All data represent mean ± S.D. of 3 independent experiments. Data were analyzed using PrismGraph with student's t-test. * indicates p-values <0.05.

The sublethal concentration of VPA (5 mM), previously shown to modulate cell surface marker expression on surviving γδ T-cells [15], induced IL-4δ_13_ and IL-4Rα as shown in a representative dot plot in Figure 2A, but no significant IFN-γ expression in Vδ2 T-cells. Results of 3 experiments are summarized in Figure 2B. These results prompted us to study a possible intracellular co-localization of IL-4δ_13_ and IL-4Rα by ImageStream cytometry. However, we failed to detect a significant degree of specific co-localization of IL-4δ_13_ and IL-4Rα (Figure 2D). Further analysis of mRNA levels for IFNγ and full-length IL-4 revealed opposite effects of VPA pretreatment, as expression levels of IFN-γ mRNA were up-regulated whereas those of mature IL-4 were down-regulated in Vδ2 T-cells (Figure 2C). This is not in contrast to the observed up-regulation of IL-4δ_13_ at the protein level, as it is known that IL-4δ_47_ (an alternative splice variant with a 47 bp deletion) also acts as a naturally occurring IL-4 antagonist [21].

### Differential changes in secretory versus non-secretory forms of IL-4

**Figure 3:**
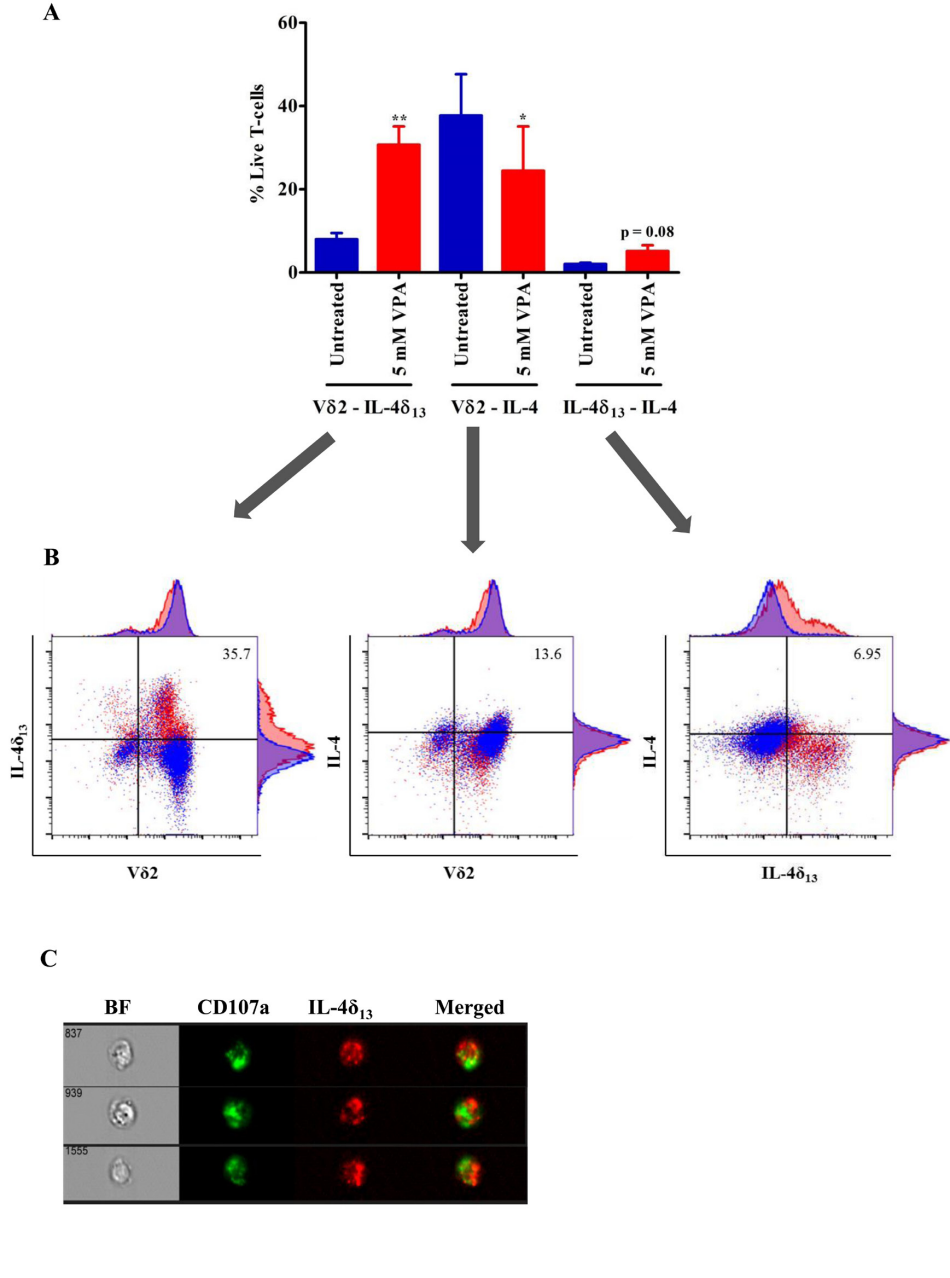
Analysis of non-secretory and mature form of IL-4 in γδ T-cells (**A**) Short-term zoledronate-stimulated γδ T-cells were treated with 5 mM VPA for 24 hrs. Afterwards, cells were harvested and flow cytometry was performed after staining with PE-IL4δ_13_ (clone 8D4-8), FITC-Vδ2 and Brilliant violet 605-IL-4 (clone MP4-25D2) and gating on live/dead fixable far-red dye negative cells. 4 independent experiments were showing mean ± S.E. Samples were acquired on BD LSR Fortessa and analyzed using FlowJo data analysis software. Statistical significance is shown by *or ** for p-values <0.05 or <0.01, respectively. (**B**) Representative dot plots show overlay of untreated (blue color) γδ T-cells with 5 mM VPA (red color) treated cells expressing respective antibody staining. Numbers represented in dot plot indicate percentage of expressing population after 5 mM VPA treatment. (**C**) In a very similar approach to Figure 2C, ImageStream analysis was performed to analyze possible localization of CD107a and IL-4δ_13_. Live/dead far red dye negative 5 mM VPA-treated γδ T-cells stained with FITC-CD107a and PE-IL-4δ_13_, are shown in single channels and in merged image, BF stands for bright field. Three representative cells from three independent experiments are shown.

In view of the reported down-regulation of full length IL-4 transcript by the IL-4δ_47_ isoform [21], we tested whether IL-4δ_13_ can also act as IL-4 antagonist. To address this question, we used the anti-IL-4 antibody clone MP4-25D2, which specifically detects the mature form of IL-4 [17]. In line with mRNA regulation (Figure 2C), we found a decrease in the intracellular expression of the mature form of IL-4 in Vδ2 T-cells after treatment with 5 mM VPA (Figure 3A, middle panel). Along with significant increase in IL-4δ_13_ expressing Vδ2 T-cells (Figure 3A, left panel), a slight increase in co-expression of both IL-4 and IL-4δ_13_ was observed after 5 mM VPA treatment (p=0.08; Figure 3A, right panel). The results presented in Figure 3A summarize three independent experiments. Representative dot plots plus histograms from a representative individual experiment (Figure 3B) clearly illustrate the expression of IL-4δ_13_ being associated with low TCR expression on Vδ2 T-cells while expression of mature IL-4 expression being associated with high level TCR expression on Vδ2 T-cells.

We further addressed the subcellular localization of IL-4δ_13_ by a combined confocal and flow cytometry approach. Interestingly, ImageStream analysis of IL-4δ_13_ consistently showed distinct punctuated localization in cytoplasmic vacuoles of Vδ2 T-cells which, however, failed to co-localize with CD107a (LAMP-1; lysosomal-associated membrane protein-1 and a marker for degranulation) (Figure 3C) or CD63 (a marker for intracellular vesicles; data not shown). Taken together, secretory and non-secretory forms of IL-4 expression seem to antagonize each other at the protein level. It may also be associated with the regulation of TCR surface expression on Vδ2 T-cells and distinct subcellular localization.

### Cell death pathway inhibitors modulate IL-4δ_13_ induction and histone modification

**Figure 4:**
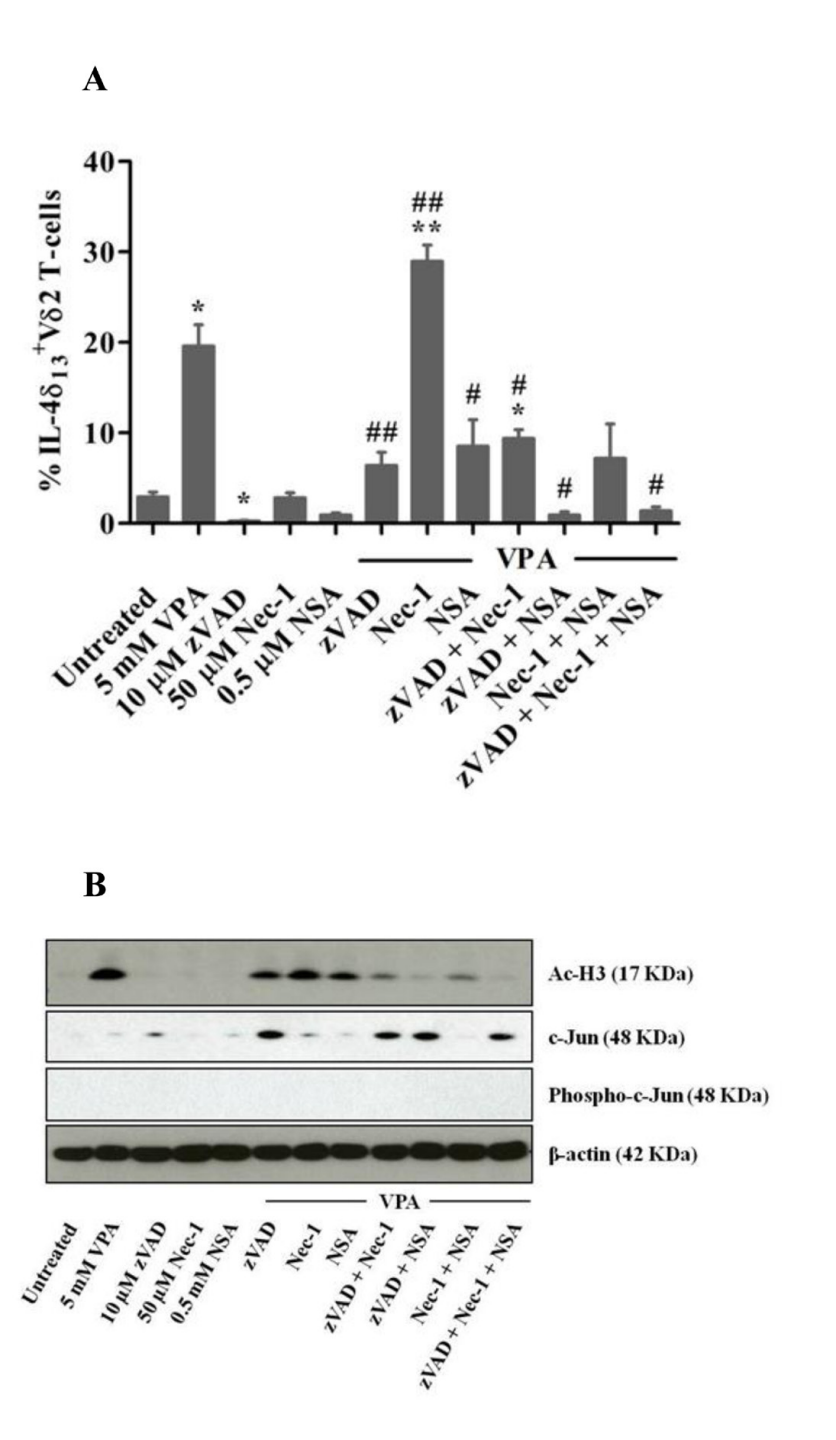
Modulation of IL-4δ_13_, c-Jun expression and histone H3 acetylation in γδ T-cells in response to VPA and cell death inhibitors γδ T-cells obtained from zoledronate-stimulated PBMC were pre-treated for 30 min with zVAD, or for 2 hrs with Nec-1 or NSA, or were left untreated. Thereafter, VPA was added where indicated. (**A**) After 24 hrs, intracellular IL-4δ_13_ expression was determined by FACS in gated Vδ2 T-cells. Dead cells were excluded based on live/dead fixable far-red dye staining. Data represents mean ± S.E. of 3 independent experiments. Data were analyzed using PrismGraph with student's t-test. p-values <0.05 were considered statistically significant and are displayed as *or ** for p-values <0.05 or <0.01 (in relation to untreated medium control) and as # or ## for p-values <0.05 or <0.01 (in relation to 5 mM VPA treatment). (**B**) After 24 hrs, cells were lysed and 10 μg protein was subjected to western blot analysis for H3K9Ac (top panel), c-Jun (upper middle panel), phospho-c-Jun (lower middle panel) and β-actin (lower panel). One representative out of three experiments is shown.

IL-4δ_13_ is known to be associated with induction of cell death [16]. Also, VPA triggers diverse apoptotic and non-apoptotic cell death pathways [22, 23]. We studied the effect of apoptosis (pan-caspase) inhibitor zVAD and inhibitors of programmed necrosis necrostatin-1 (Nec-1) and necrosulfonamide (NSA) on VPA-induced cell death and IL-4δ_13_ induction. Inhibitor concentrations known to block apoptosis and necroptosis in established cell systems [24, 25] only moderately reduced cell death of VPA-treated Vδ2 T-cells using three different read-outs (Supplemental Figure 1). Exogenous supply of IL-4 has been shown to prevent T-cell apoptosis and to modulate cellular functions [26, 27]. However, exogenous IL-4 did not rescue VPA-treated Vδ2 T cells from cell death (data not shown). zVAD, Nec-1 and NSA in combination prevented IL-4δ_13_ induction in VPA-treated live Vδ2 T-cells (Figure 4A), but not Nec-1 alone. Next, we analyzed modulation of global histone acetylation marker H3K9Ac by VPA and cell death inhibitors (Figure 4B, upper panel). We also analyzed Ac-H2Blys5, HDAC-1, HDAC-2 histone modifications; this resulted in similar patterns of protein expression as presented for H3K9Ac (data not shown). However, HDAC2 expression was inconsistent in independent experiments with several donors (data not shown). Correlating with the modulation of IL-4δ_13_ expression, treatment with zVAD and Nec-1 (but not NSA) slightly reduced H3K9 acetylation induced by VPA. Combination of any two inhibitors further reduced acetylation, while complete loss of histone acetylation marks was observed when all three inhibitors were combined together. Thus, cell death inhibitors directly regulate IL-4δ_13_ and global H3K9 acetylation.

### Interplay of IL-4δ_13_ with c-Jun expression and histone acetylation

**Figure 5:**
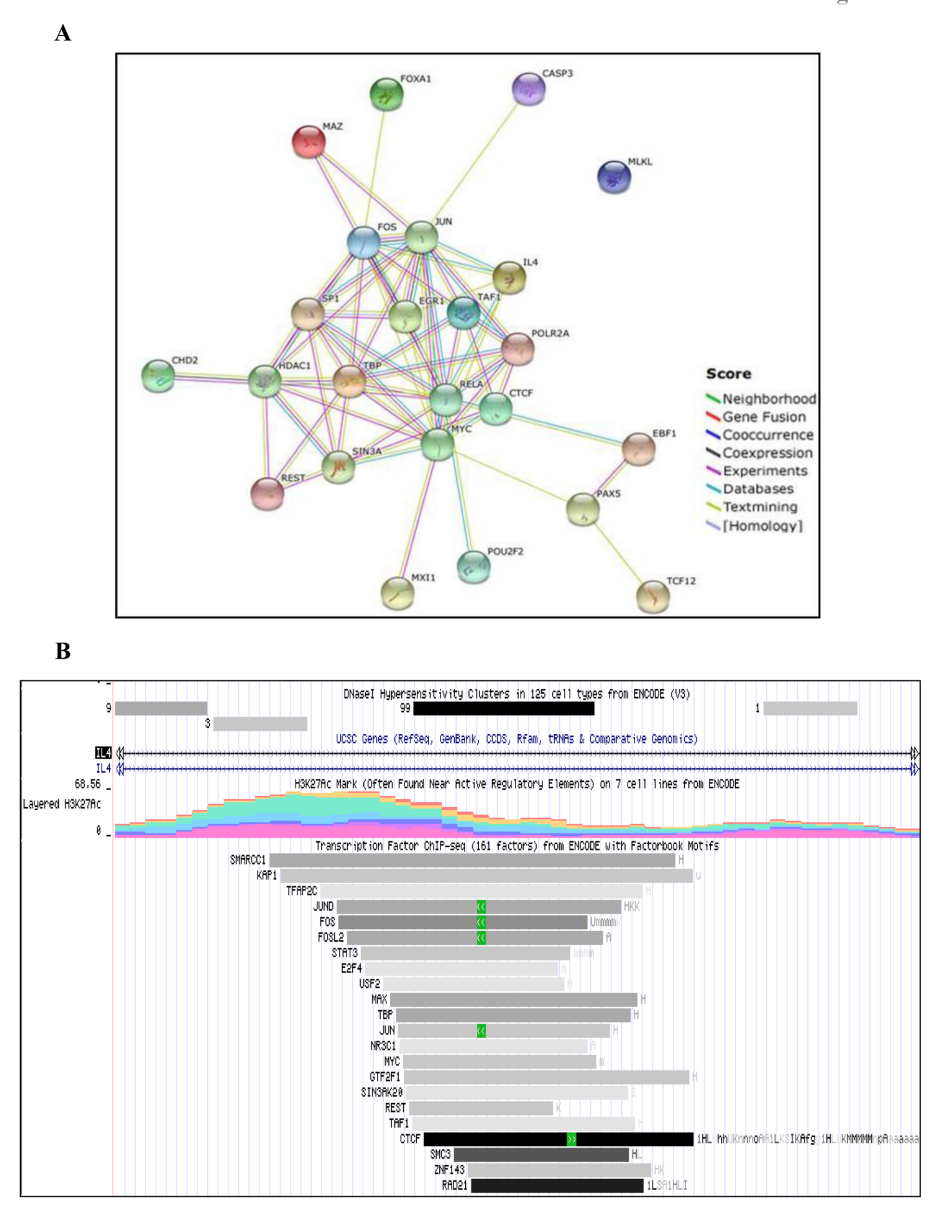
Bioinformatic analysis of commonly occurring transcription factors in *in silico* IL-4 regulatory network (**A**) Sets of commonly occurring transcription factors (see Supplementary Table 1) were further analyzed for protein-protein interaction by STRING database. The absence of lines linking MLKL to other factors indicates that no such interactions have so far been described in the literature. (**B**) Bioinformatic analysis was done using the available ChIP-seq ENCODE transcription factors database. Representative image shows the number of transcription factors including c-Jun, found in IL-4 genomic region that are associated H3K27Ac mark.

H3K9 plays an important dual role in gene regulation. Acetylation of H3K9 correlates with active promoter and gene transcription, while methylation is associated with gene silencing [28]. Based on this information, we analyzed a number of transcription factors involved in regulation of *IL-4*, *HDAC1* (the target of VPA), *MLKL* (the target for NSA) and *CASP3* (the target for zVAD) genes, which had been identified from ChIP-seq experiments by the ENCODE project (http://genome.ucsc.edu/ENCODE/index.html) [29]. A list of transcription factors commonly ranked to be involved in the regulation of all four genes is listed in Supplementary Table 1. String database analysis of this unique set of transcription factors revealed potential protein-protein interactions (Figure 5A).

Our bioinformatic analysis of *IL-4* gene using UCSC genome browser revealed putative binding sites of a subset of transcription factors, which are associated with H3K27Ac marks often found near active regulatory elements (Figure 5B). c-Jun was one of the unique transcription factors associated with all four genes. Hence, we chose to validate its role in this network at the protein level (Figure 4B, middle panel). HDACi have been reported to suppress the induction of c-Jun [30]. In line, we did not observe induction of c-Jun by VPA alone, but there was a striking up-regulation in the additional presence of zVAD. Whenever zVAD was present (together with VPA), enhanced c-Jun expression was observed, while NSA or Nec-1 had no effect (Figure 4B, middle panel). The stimulatory effect of zVAD on c-Jun expression is in agreement with previous reports [31]. Of note, phosphorylation of c-Jun was not detected, possibly due to the selected time point or reflecting the dispensability of c-Jun phosphorylation for T-cell proliferation [30]. Most importantly, however, we found that the combination of zVAD, NSA and Nec-1 led to a complete loss of VPA-induced H3 acetylation mark (Figure 4B, upper panel) which correlated with strongest inhibition of IL-4δ_13_ induction (Figure 4A) but marked c-Jun expression (Figure 4B, middle panel). The absence of any histone mark observed here in the presence of a combination of cell death inhibitors has not been previously reported in the context of blockade of apoptosis [33]. Thus, our results revealed a potential interrelation between epigenetic modification (H3K9Ac), intracellular cytokine (IL-4δ_13_) induction, transcription factor (c-Jun) expression and regulation by cell death inhibitors.

**Figure 6:**
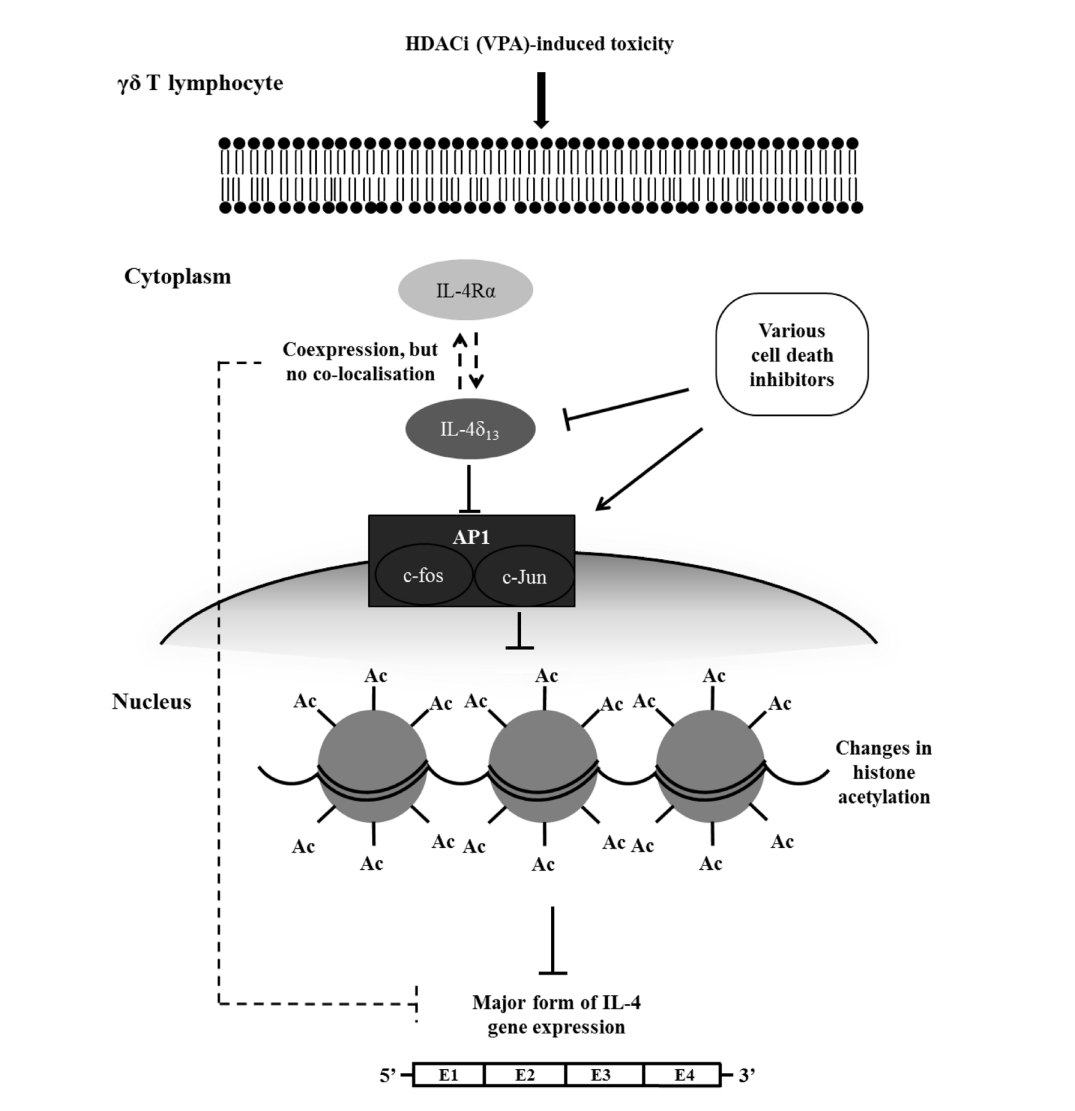
Schematic diagram of proposed regulatory interplay between VPA-induced toxicity, induction of IL-4δ_13_ and c-Jun, histone acetylation, and modulation by cell death inhibitors When human γδ T-cells are treated with VPA at sublethal concentrations, non-secreted form of IL-4 (described as IL-4δ_133_) and intracellular IL-4Rα are induced, which however fail to directly interact. Cell death inhibitors modulate c-Jun transcription factor and IL-4δ_133_ expression as well as active histone acetylation global mark. In parallel to induced protein expression of IL-4δ_133_ and IL-4Rα, this signaling cascade might down-regulate gene expression of the major form of IL-4.

## DISCUSSION

VPA is widely used as a mood stabilizer and anti-epileptic drug. Apart from its recently identified inhibitory activity on histone deacetylase, it has been shown to directly target Gamma Amino Butyrate (GABA) transaminobutyrate and ion channels [34]. VPA is also tested in various clinical trials for cancer therapy (NCT01182285 and NCT00302159; http://clinicaltrials.gov) [13]. The clinical dosage varies substantially in different cancer types and with the treatment protocol, depending on whether is used as monotherapy or in combination therapy [35-39]. Under *in vitro* conditions, VPA has been shown to modulate diverse functional responses at concentrations ranging from 0.5 mM to 10 mM [40-42].

We previously reported that HDAC inhibition by VPA modulates the expression of certain cell surface proteins [15]. Apart from VPA, EGCG (HAT and DNMT inhibitor), TSA (HDAC inhibitor) and decitabine (DNMT inhibitor) have been reported to modulate expression of intracellular cytokines and transcription factors [18, 19]. Valapour *et al*, (2002) [19] reported that IL-4 production by activated peripheral blood T-cells is enhanced by TSA. Hence, we analyzed intracellular expression of IL-4 in human γδ T-cells upon treatment with inhibitors for HDAC and DNMT by flow cytometry following appropriate exclusion of dead cells. The IL-4 protein can be expressed in different isoforms. In our experiments, we used different antibodies to monitor IL-4 expression, specifically the 8D4-8 clone, which detects the unglycosylated, non-secreted form of IL-4 (IL-4δ_13_, 13 bp deleted form) and the MP4-25D2 clone which detects the mature form of IL-4 [16, 17, 20]. Treatment with VPA, EGCG and TSA alone or in combination with VPA enhanced the expression of IL-4δ_13_. “Toxic” concentrations of VPA (5 mM) significantly induced expression of IL-4δ_13_ and decreased the expression of mature IL-4, but did not modulate IFN-γ cytokine. Increase in IL-4δ_13_ was associated with increase in intracellular IL-4R (Figure 2A-2B). On the contrary, the mature form of IL-4 mRNA expression was down regulated. It is already known that IL-4δ_2_ (an alternative splice variant with 47 bp deletion) acts like a naturally occurring human IL-4 antagonist [21]. We failed to reveal significant interaction of IL-4δ_13_ with the chain of IL-4R, which is in contrast to IL-4δ_2_ signaling pathway [21]. We also attempted to characterize the subcellular localization of IL-4δ_13,_ but we observed only partial co-localization with CD107a and CD63. It seems that IL-4δ_13_ is neither secreted in exosomes nor undergoes lysosomal degradation and is packaged in specialized vesicles.

Different signaling pathways are involved in regulation of cell death. Such regulated cell death may trigger inflammation and induces signaling cascades [43]. The role of distinct signaling pathways can be addressed through specific inhibitors. Previous reports already showed that the expression of IL-4δ_13_ is associated with programmed cell death, notably apoptosis [16, 20]. In recent years, however, it has been realized that caspase-independent non-apoptotic cell death pathways (programmed necrosis) are equally important in the regulation of cell death and survival [43]. VPA is also known to execute diverse cell death mechanisms, other than apoptosis in multiple myeloma and neuronal cells [22, 23]. In our experiments, we used cell death inhibitors to explore a potential role of distinct cell death pathways using pan-caspase inhibitor zVAD (to block apoptosis) and additionally Nec1, NSA (to block programmed necrosis) (Figure 4A-4B). Pre-treatment of γδ T-cells with zVAD, Nec1 and NSA substantially modulated VPA-induced IL-4δ_13_ expression. It should be noted that using various experimental read-out systems, these inhibitors when used alone or even in combination did not completely rescue VPA-treated cells from cell death. Similarly, exogenous IL-4 did not prevent VPA-induced cell death of γδ T-cells. A possible explanation might be that VPA induces diverse cell death pathways, some of which might not be influenced by the inhibitors used here. Although not analyzed in detail, VPA might have induced a ‘mixed’ type of cell death in γδ T-cells.

In the same experimental set up, we analyzed the induction of histone acetylation. In line with the modulation of IL-4δ_13_ expression, treatment with zVAD, Nec1 and NSA modulated H3K9Ac leading to complete loss of histone acetylation marks induced by VPA. Furthermore, location of c-Jun transcription factor interaction site on *IL-4* gene was associated with H3K27 marks (Figure 5B). Our network analysis was validated at the protein level by expression of c-Jun transcription factor (Figures 4B and 5A). Previous reports suggested that HDAC inhibitors block c-Jun transcription [30], while c-Jun phosphorylation is not required for T-cell proliferation or differentiation, only for thymocyte apoptosis [32]. Our protein analysis by western blot revealed very similar results in terms of HDAC inhibitor treatment and phosphorylation of c-Jun.

The characteristic features of apoptosis are histone degradation, chromatin condensation and DNA fragmentation. Absence of any histone mark has not been reported with blockade of apoptosis [33]. Available reports showed that serine proteases like granzyme A could induce histone proteolysis and increase accessibility of cellular DNA to nucleases [44]. Also, it has been suggested that the apoptotic effects of HDACi are predominantly elicited through their impact on gene expression rather than on histone acetylation [45]. However, IL-4δ_13_ may act as survival signal to cells undergoing differentiation. It would be interesting to further investigate such a potential Th2 bias effect associated with histone modification in γδ T-cells from neonates and adult individuals. Moreover, in an autoimmune encephalitis mouse model, Lv and coworkers observed a role of non-toxic doses of VPA in maintaining immune homeostasis through induction of apoptosis in activated T-cells [46]. But, evidence for an induction of the non-secreted form of IL-4 in any disease, except HIV pathogenesis, is completely missing. Primate models are the most suitable experimental system for both HIV disease and human γδ T-cell research, because they share the Vγ9Vδ2 TCR required for recognition of microbial pyrophosphates [1, 2]. Interestingly, evolutionary studies in primates of genes involved in HIV pathogenesis have also identified IL-4 as one of the proteins undergoing diverse positive selection [47, 48]. As a result of such evolutionary processes, IL-4δ_13_ is expressed at the protein level, but the cellular and molecular significance have not been defined.

Our present study depicts the potential role of c-Jun transcription factor in correlation with IL-4δ_13_ expression and histone acetylation (Figure 6) and previous reports show that various isoforms of IL-4 share potential crosstalk regulating mature IL-4 expression. Future experiments will focus on analyzing functional genomic features of the 13 bp deletion in comparison to IL-4δ_47_ and full length form of IL-4 under various *in vitro* conditions. Additional experiments will be performed with γδ T-cell and tumor cell co-cultures to study the role of VPA-generated intracellular cytokines. Taking all evidence together, the expression of novel IL-4δ_13_ may emerge as a ‘combined marker’ for cell death, epigenetic modification and T-cell differentiation.

## MATERIALS AND METHODS

### Cell culture

PBMC were isolated from leukocyte concentrates obtained from healthy donors (Department of Transfusion Medicine, UKSH). Informed consent was obtained from all donors, and the study was approved by the local Ethics Committee (D405/10). Cell cultures were set up in RPMI-1640 medium supplemented with 10 % heat inactivated FCS and antibiotics (Biochrom). PBMC were stimulated with 2.5 μM zoledronate (Novartis) in the presence of 50 IU/ml IL-2 (Novartis). IL-2 was repeatedly added every 2 days. After 12 days, such cultures routinely contained >90 % Vγ9Vδ2-expressing γδ T-cells. αβ T-cell lines were generated by stimulating PBMC with a mixture of staphyloccal enterotoxins (1 ng/ml each) (SEA, SEC1, SED, SEE; hereafter referred as SE-Mix; Toxin Technology, Florida, USA) for 7 days. αβ T-cells or γδ T-cells were cultured at 1×10^6^ per ml for 24 hrs in the absence or presence of Valproic acid (VPA), Trichostatin A (TSA), or 5-Aza-2′-deoxycytidine (Decitabine) (all from Sigma-Aldrich). Where indicated, γδ T-cells were first pretreated with cell death inhibitors Z-Val-Ala-DL-Asp-fluoromethylketone (zVAD, Bachem), Necrosulfonamide (NSA, Merck Chemicals), or Necrostatin-1 (Nec-1, Sigma-Aldrich). While zVAD is a pan-caspase inhibitor which blocks apoptosis, Nec-1 inhibits RIPK1, and NSA MLKL, thereby blocking programmed necrosis/necroptosis [24, 25].

### Flow cytometry

The following reagents and anti-human antibodies were used: anti-IL-4 PE (clone 8D4-8 detecting non-secreted IL-4δ_13_; BD Biosciences, Heidelberg, Germany), anti-IL-4 Brilliant Violet 605 (clone MP4-25D2 detecting mature form of IL-4; BioLegend, Fell, Germany), anti-IFN-γ PE (clone 4S.B3; BD Biosciences), anti-Vδ2 FITC (clone IMMU389; Beckman Coulter, Krefeld, Germany), anti-Vδ2 PerCP (clone B6; BioLegend), anti-human CD107a FITC (clone H4A3; BioLegend), rabbit anti-human IL-4Rα (sc-684; Santa Cruz Biotechnology, Heidelberg, Germany), Alexa Fluor 488-conjugated donkey anti-rabbit IgG (H+L) (Life Technologies GmbH, Darmstadt, Germany), Annexin V-FITC (MabTag, Friesoythe, Germany), PI (Serva, Heidelberg, Germany). For dead cell exclusion, live/dead fixable far-red dead cell stain kit (Life Technologies) was used. After respective treatment with the epigenetic modifiers and/or cell death inhibitors, monensin was added to the cultures of γδ T-cells for last 4 hrs. Thereafter, cells were harvested and stained with live/dead fixable dye according to manufacturer's protocol (Life Technologies). Further, cells were stained for Vδ2 surface marker and intracellularly for IL-4δ_13_, IL-4, IL-4Rα, and IFN-γ following treatment with fixation/permeabilization solution kit (BD Biosciences). Flow cytometry was done by gating on live cells. Samples were acquired either on a FACSCalibur flow cytometer (BD Biosciences) and analyzed using the CellQuest Pro software (BD Biosciences) or on a LSR Fortessa (BD Biosciences) and analyzed using the FlowJo software (FlowJo LLC, Ashland Or, USA).

### ImageStream analysis

ImageStream flow cytometry was performed to study possible co-localization of IL-4δ_13_ with IL-4Rα, and CD107a. Cells were stained for IL-4δ_13_ and IL-4Rα or CD107a together with live/dead fixable dye and acquired on ImageStreamX Mark II imaging flow cytometer (Merck Millipore). Images (60X) and statistics were processed by using IDEAS software (version 6.0, Amnis) applying co-localization wizard [49].

### Quantification of mRNA expression

mRNA expression was quantified by real time PCR specific for the major form of human IL-4 and IFN-γ genes. After 24 hrs of VPA treatment, RNA was isolated from γδ T-cells using the peqGOLD TriFast Isolation procedure (Peqlab, Erlangen, Germany). RNA (200 ng) was transcribed into cDNA using the cDNA synthesis kit (AmpTec, Hamburg, Germany). For PCR amplification, *IL-4* gene: forward primers (5′-GCCACCATGAGAAGGACACT-3′) and reverse primers (5′-ACTCTGGTTGGCTTCCTTCA-3′); and *IFN-γ* gene: forward primers (5′-TCAGCTCTGCATCGTTTTGG-3′) and reverse primers (5′-GTTCCATTATCCGCTACATCTGAA-3′) were used at 60°C. qPCR data were analyzed according to the ΔΔCt method using the mean Ct value of the housekeeping genes (β-actin, β2-microglobulin and 18S). Fold changes of expression levels were calculated and the obtained values were used for further statistical analysis [50].

### Cell death analysis

Cell death analysis for VPA-induced toxicity of γδ T-cells was performed by combined annexin-V FITC/ PI staining and also additionally by ATP release using the Cell Titer-Glo Luminescent Cell Viability Assay (Promega GmbH, Mannheim, Germany). Briefly, after 24 hrs treatment of γδ T-cells with VPA and cell death inhibitors (zVAD, Nec1, NSA), cells were harvested for annexin V-FITC/ PI staining as described [15]. Cell viability using the ATP assay was analyzed according to the manufacturer's protocol. Luminescence was measured by Tecan Infinite-200 microplate reader (Tecan Group Ltd., Maennedorf, Germany).

### Immunoblotting

Western blot analysis was done on γδ T-cell lysates. Samples were run on NuPAGE 4-12 % Bis-Tris Gel (Life Technologies). Blotting was performed with Hybond C extra nitrocellulose membranes (GE Healthcare, Munich, Germany). Primary antibodies against human acetyl H3K9 (clone C5B11; Cell Signaling Technology Inc. Beverly, MA, USA), c-Jun (clone 60A8; Cell Signaling Technology), Phospho-c-Jun (clone D47G9; Cell Signaling Technology) and β-actin (clone AC-15; Sigma-Aldrich) were incubated overnight and further detected with HRP-conjugated secondary antibodies (GE Healthcare). Proteins were visualized by the enhanced chemiluminescence system (GE Healthcare).

### Bioinformatics analysis

*IL-4, CASP3, MLKL, HDAC1* genes were analyzed in UCSC Genome Browser (hg19 assembly). A list of transcription factors associated with respective genes from ChIP-seq ENCODE database with factorbook motifs were derived (see Supplemental Table 1). The unique set of transcription factors involved in regulation of all 4 genes was further analyzed using Phospho-c-Jun database for protein-protein interaction and association studies. All prediction methods, viz. neighborhood, gene fusion, co-occurance, co-expression, experiments, databases and text mining were used with high confidence score (0.700), showing direct interaction through links for each type of method as described by Szklarczyk *et al* (2015) [51].

### Statistical analysis

Data were analyzed using PrismGraph with student's t-test. p-values <0.05 were considered statistically significant and displayed as * or ** for p-values <0.05 or <0.01, respectively.

## SUPPLEMENTARY MATERIAL FIGURES AND TABLE





## Abbreviations

SE: Staphyloccal enterotoxin
TCR: T-cell receptor
VPA: Valproic acid
