# Supplementary material for: Expression of non-secreted IL-4 is associated with HDAC inhibitor-induced cell death, histone acetylation and c-Jun regulation in human gamma/delta T-cells
Source: Oncotarget. 2016 Aug 20;7(40):64743–56. doi: 10.18632/oncotarget.11462 (PMC5323112; doi:10.18632/oncotarget.11462)
Supplement: Supplementary file 1 [file oncotarget-07-64743-s001.pdf]

Expression of non-secreted IL-4 is associated with HDAC inhibitor-induced cell death, histone acetylation and c-Jun regulation in human gamma/delta T-cells

Supplementary Material

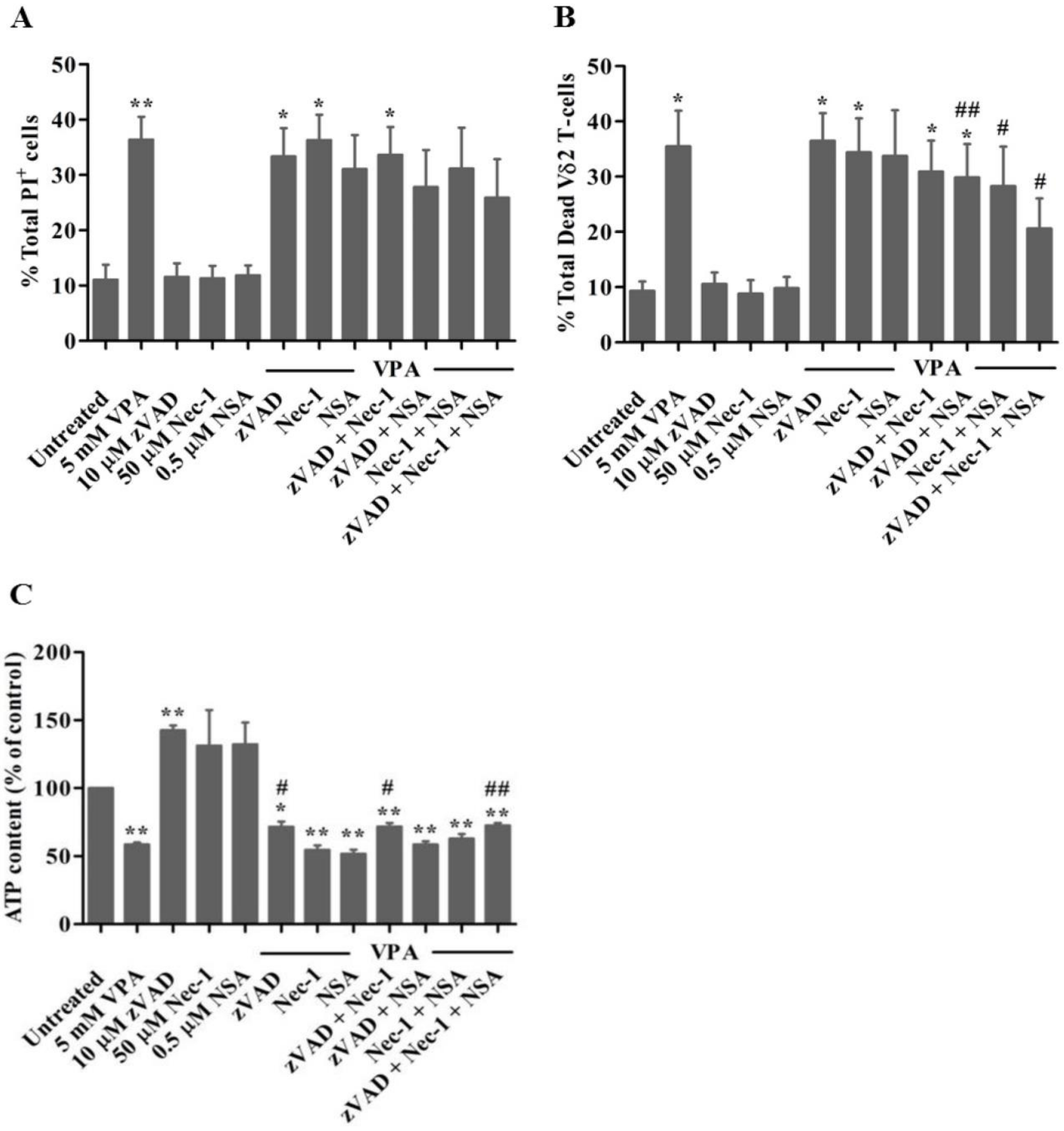

Supplemental Figure 1: **Modulation of VPA-induced γδ T-cell death by cell death inhibitors.** Human short-term γδ T-cell lines were treated for 24 hrs with VPA and cell death inhibitors (Nec-1, NSA, zVAD). (A) After treatment cells were harvested and stained with annexin-V FITC and PI. Proportions of total PI positive are indicated. (B) Similarly, treated cells were harvested and stained for Vδ2 surface marker together with live/dead fixable dye. Samples were analyzed by gating on Vδ2 marker and distinguished as live or dead based on live/dead fixable dye labelling. Data represent mean ± S.E. of 3 independent experiments. (C) Cells, treated as previously described, were harvested and the ATP content was measured by Cell Titer-Glo Luminescent Cell Viability Assay and calculated relative to the untreated cells (set as 100%). Data were analyzed using PrismGraph with student's t-test. p values <0.05 were considered statistically significant and are displayed as * or ** for p-values <0.05 or <0.01 (in relation to untreated medium control) and as # or ## for p-values <0.05 or <0.01 (in relation to 5 mM VPA treatment).

Supplementary Table 1: List of unique candidate proteins derived from STRING database analysis for protein-protein interaction and association. Corresponding information is mentioned in the Table.
