# Supplementary material for: Expression of non-secreted IL-4 is associated with HDAC inhibitor-induced cell death, histone acetylation and c-Jun regulation in human gamma/delta T-cells
Source: Oncotarget. 2016 Aug 20;7(40):64743–56. doi: 10.18632/oncotarget.11462 (PMC5323112; doi:10.18632/oncotarget.11462)
Supplement: Supplementary file 2 [file oncotarget-07-64743-s002.doc]

**Supplementary Table 1:**

List of unique candidate proteins derived from STRING database analysis for protein-protein interaction and association. Corresponding information is mentioned in the Table.

| **Sr. No.** | **Symbol of the protein** | **Name and information of protein** |
| --- | --- | --- |
| 1 | CHD2 | Chromodomain helicase DNA binding protein 2; Sequence-selective DNA-binding protein (By similarity) (1828 aa) |
| 2 | CTCF | CCCTC-binding factor (zinc finger protein); Chromatin binding factor that binds to DNA sequence specific sites. Involved in transcriptional regulation by binding to chromatin insulators and preventing interaction between promoter and nearby enhancers and silencers. Acts as transcriptional repressor binding to promoters of vertebrate c-myc gene and BAG1 gene. Also binds to the PLK and PIM1 promoters. Acts as a transcriptional activator of APP. Regulates APOA1/C3/A4/A5 gene cluster and controls MHC class II gene expression. Plays an essential role in oocyte and preimplantation embryo development (727 aa) |
| 3 | EBF1 | Early B-cell factor 1; Transcriptional activator which recognizes variations of the palindromic sequence 5'-ATTCCCNNGGGAATT-3' (By similarity) (591 aa) |
| 4 | EGR1 | Early growth response 1; Transcriptional regulator. Recognizes and binds to the DNA sequence 5'-CGCCCCCGC-3'(EGR-site). Activates the transcription of target genes whose products are required for mitogenesis and differentiation (543 aa) |
| 5 | FOS | FBJ murine osteosarcoma viral oncogene homolog; Nuclear phosphoprotein which forms a tight but non- covalently linked complex with the JUN/AP-1 transcription factor. In the heterodimer, c-fos and JUN/AP-1 basic regions each seems to interact with symmetrical DNA half sites. Has a critical function in regulating the development of cells destined to form and maintain the skeleton. It is thought to have an important role in signal transduction, cell proliferation and differentiation (380 aa) |
| 6 | FOXA1 | Forkhead box A1; Transcription activator for a number of liver genes such as AFP, albumin, tyrosine aminotransferase, PEPCK, etc. Interacts with the cis-acting regulatory regions of these genes (472 aa) |
| 7 | JUN | Jun oncogene; Transcription factor that recognizes and binds to the enhancer heptamer motif 5'-TGA[CG]TCA-3' (331 aa) |
| 8 | MAZ | MYC-associated zinc finger protein (purine-binding transcription factor); May function as a transcription factor with dual roles in transcription initiation and termination. Binds to two sites, ME1a1 and ME1a2, within the c-myc promoter having greater affinity for the former. Also binds to multiple G/C-rich sites within the promoter of the Sp1 family of transcription factors (493 aa) |
| 9 | MXI1 | MAX interactor 1; Transcriptional repressor. MXI1 binds with MAX to form a sequence-specific DNA-binding protein complex which recognizes the core sequence 5'-CAC[GA]TG-3'. MXI1 thus antagonizes MYC transcriptional activity by competing for MAX (295 aa) |
| 10 | MYC | v-myc myelocytomatosis viral oncogene homolog (avian); Participates in the regulation of gene transcription. Binds DNA in a non-specific manner, yet also specifically recognizes the core sequence 5'-CAC[GA]TG-3'. Seems to activate the transcription of growth-related genes (454 aa) |
| 11 | PAX5 | Paired box 5; May play an important role in B-cell differentiation as well as neural development and spermatogenesis. Involved in the regulation of the CD19 gene, a B-lymphoid-specific target gene (391 aa) |
| 12 | POLR2A | Polymerase (RNA) II (DNA directed) polypeptide A, 220kDa; DNA-dependent RNA polymerase catalyzes the transcription of DNA into RNA using the four ribonucleoside triphosphates as substrates. Largest and catalytic component of RNA polymerase II which synthesizes mRNA precursors and many functional non-coding RNAs. Forms the polymerase active center together with the second largest subunit. Pol II is the central component of the basal RNA polymerase II transcription machinery. It is composed of mobile elements that move relative to each other (1970 aa) |
| 13 | POU2F2 | POU class 2 homeobox 2; Transcription factor that specifically binds to the octamer motif (5'-ATTTGCAT-3'). Regulates transcription in a number of tissues in addition to activating immunoglobulin gene expression. Modulates transcription transactivation by NR3C1, AR and PGR. Isoform 5 activates the U2 small nuclear RNA (snRNA) promoter (463 aa) |
| 14 | RELA | v-rel reticuloendotheliosis viral oncogene homolog A (avian); NF-kappa-B is a pleiotropic transcription factor which is present in almost all cell types and is involved in many biological processed such as inflammation, immunity, differentiation, cell growth, tumorigenesis and apoptosis. NF- kappa-B is a homo- or heterodimeric complex formed by the Rel-like domain-containing proteins RELA/p65, RELB, NFKB1/p105, NFKB1/p50, REL and NFKB2/p52 and the heterodimeric p65-p50 complex appears to be most abundant one. The dimers bind at kappa-B sites in the DNA of their target genes (551 aa) (Homo sapiens) |
| 15 | REST | RE1-silencing transcription factor; Transcriptional repressor which binds neuron-restrictive silencer element (NRSE) and represses neuronal gene transcription in non-neuronal cells. Restricts the expression of neuronal genes by associating with two distinct corepressors, mSin3 and CoREST, which in turn recruit histone deacetylase to the promoters of REST-regulated genes. Mediates repression by recruiting the BHC complex at RE1/NRSE sites which acts by deacetylating and demethylating specific sites on histones, thereby acting as a chromatin modifier (1097 aa) |
| 16 | SIN3A | SIN3 homolog A, transcription regulator (yeast); Acts as a transcriptional repressor. Interacts with MXI1 to repress MYC responsive genes and antagonize MYC oncogenic activities. Also interacts with MAD-MAX heterodimers by binding to MAD. The heterodimer then represses transcription by tethering SIN3A to DNA. Acts as a corepressor for REST (By similarity) (1273 aa) |
| 17 | SP1 | Sp1 transcription factor; Transcription factor that can activate or repress transcription in response to physiological and pathological stimuli. Binds with high affinity to GC-rich motifs and regulates the expression of a large number of genes involved in a variety of processes such as cell growth, apoptosis, differentiation and immune responses. Highly regulated by post-translational modifications (phosphorylations, sumoylation, proteolytic cleavage, glycosylation and acetylation). Binds also the PDGFR- alpha G-box promoter (785 aa) |
| 18 | TAF1 | TAF1 RNA polymerase II, TATA box binding protein (TBP)-associated factor, 250kDa; Largest component and core scaffold of the TFIID basal transcription factor complex. Contains novel N- and C-terminal Ser/Thr kinase domains which can autophosphorylate or transphosphorylate other transcription factors. |
| 19 | TBP | TATA box binding protein; General transcription factor that functions at the core of the DNA-binding multiprotein factor TFIID. Binding of TFIID to the TATA box is the initial transcriptional step of the pre- initiation complex (PIC), playing a role in the activation of eukaryotic genes transcribed by RNA polymerase II. Component of the transcription factor SL1/TIF-IB complex, which is involved in the assembly of the PIC (preinitiation complex) during RNA polymerase I-dependent transcription. The rate of PIC formation probably is primarily dependent on the rate of association of SL1 (339 aa) |
| 20 | TCF12 | Transcription factor 12; Binds specifically to oligomers of E-box motifs. May play important roles during development of the nervous system as well as in other organ systems (706 aa) |
